# One-hole split endoscope versus unilateral biportal endoscopy for lumbar spinal stenosis: a retrospective propensity score study

**DOI:** 10.1186/s13018-024-04743-7

**Published:** 2024-04-22

**Authors:** Tusheng Li, Qiang Jiang, Wei Zhong, Tengyue Zhu, Zhengcao Lu, Yu Ding

**Affiliations:** 1 Orthopedics of TCM Senior Department, The Sixth Medical Center of PLA General Hospital, 6 Fucheng Road, Haidian District, Beijing, 100048 People’s Republic of China; 2 The Second School of Clinical Medicine, Southern Medical University, Guangzhou, People’s Republic of China; 3 School of Medicine, South China University of Technology, Guangzhou, People’s Republic of China

**Keywords:** One-hole split endoscope, Unilateral biportal endoscopy, Lumbar spinal stenosis, Minimally invasive surgery, Decompression

## Abstract

**Background:**

The one-hole split endoscopy (OSE) was first proposed and clinically applied in China in 2019. The aim of this study was to compare the clinical efficacy of one-hole split endoscopy (OSE) and unilateral biportal endoscopy (UBE) for treating lumbar spinal stenosis (LSS).

**Methods:**

One hundred sixty patients with LSS who met the inclusion from November 2020 to August 2022 were analyzed and divided into OSE and UBE groups. The propensity score matching (PSM) method was used to adjust the imbalanced confounding variables between the two groups. After matching, surgical outcomes were recorded, and clinical data, including functional scores and imaging findings, were compared. Functional scores included the visual analog scale of leg pain (VAS-LP) and back pain (VAS-BP), the Japanese Orthopedic Association score (JOA), and the Oswestry Disability Index (ODI). Imaging data included dural sac cross-sectional area (DCSA), lumbar range of motion (ROM), and sagittal translation (ST).

**Results:**

After PSM, 104 LSS patients were included in the study, and all covariates were well-balanced between the two groups. Among the matched patients, the OSE showed advantages over the UBE regarding operative time (62.42 ± 4.86 vs. 68.96 ± 4.56) and incision length (2.30 ± 0.14 vs. 2.70 ± 0.15) (*P* < 0.001). However, differences between the two groups in intraoperative blood loss, hospital length of stay, and complication rates were not statistically significant (*P* > 0.05). There was no statistically significant difference regarding VAS-BP, VAS-LP, JOA, and ODI between the two groups (*P* > 0.05). However, all clinical and functional scores significantly improved postoperatively (*P* < 0.05). Postoperative DCSA of both groups was significantly found to be improved (*P* < 0.05), ROM and ST remained within the normal range, and no cases of lumbar instability were recorded. According to the modified MacNab criteria, the excellent and good rates in the OSE and UBE groups were 94.23% and 90.38%, respectively, with no statistically significant difference (*P* = 0.713).

**Conclusion:**

OSE is an alternative technique to UBE for the treatment of LSS, with similar satisfactory clinical outcomes, shorter operative time, and smaller incision length. Further studies are needed for long-term efficacy.

## Introduction

With a growing elderly population, lumbar spinal stenosis (LSS) has gained more attention as a common spinal degenerative disease in middle-aged and elderly [1]. LSS is characterized by reduced spinal canal volume due to pathological factors such as the proliferation of the facet joints, hypertrophy of the ligamentum flavum, and protrusion of the intervertebral disc [2]. In clinical practice, spinal canal narrowing can compress the cauda equina and/or nerve roots, developing severe conditions such as neurogenic intermittent claudication, lower limb pain, and numbness [2, 3]. Currently, for those LSS patients not responding to conservative treatment, surgical intervention is inevitable [4]. Traditional open surgery is the classic procedure for the treatment of LSS, requiring extensive intraoperative stripping of the paravertebral muscles and large-scale resection of the vertebral plate and facet joints, which can lead to postoperative complications such as intractable low back pain, muscle denervation, and lumbar spine instability [3, 5]. Recently, with the rapid development of minimally invasive techniques, spinal endoscopic surgery has received increasing attention for LSS treatment [6]. Unilateral biportal endoscopy (UBE), due to its advantages of flexibility, minimal trauma, and fast recovery, has become one of the mainstream surgical procedures treating LSS [2, 7]. The development of the UBE technique has effectively addressed issues such as narrow channels and limited surgical instruments during single-channel endoscopic and traditional microsurgical decompression procedures [8].

The one-hole split endoscopy (OSE) was first proposed and clinically applied in China in 2019. Like the UBE technique, the OSE consists of an observation and a working channel. Compared with the UBE technique, two channels in the OSE are located within the same soft incision, allowing independent and free rotation and swinging without the limitations of fixed channels [9, 10]. It does not have the limitation of forming a “V” angle, and the working and observation channels cooperate more effectively in the same direction, with fewer visual blind spots. OSE is a relatively new technique that integrates the technical concepts of UBE and coaxial endoscopy, and is anticipated to explore a balance between more efficient decompression, less trauma, and improved flexibility for minimally invasive spinal endoscopy. LSS is a surgical indication for the OSE technique, nevertheless, the clinical efficacy, feasibility and operational points still need to be further investigated and clarified due to the lack of systematic literature reports. The current study retrospectively analyzed the clinical outcomes of two techniques for the treatment of LSS, with the aim of investigating the clinical advantages and safety of the OSE technique.

## Materials and methods

### Study design and patients

This study was a single-center retrospective clinical study approved by the Ethics Committee of the Sixth Medical Center of the PLA General Hospital (No. HZKY-PJ-2023-39), and written informed consent was preoperatively obtained from all participants. From November 2020 to August 2022, 160 LSS patients who underwent OSE or UBE therapy, including 62 patients who received OSE treatment (OSE group) and 98 patients who received UBE treatment (UBE group), were included.

Table 1 provides more details regarding inclusion and exclusion criteria. Due to the imbalance of confounding factors in the two groups during the study, propensity score matching (PSM) (caliper value set at 0.02) was performed to balance the effects of confounding covariates on clinical outcomes. The propensity score for each patient was calculated as the probability of receiving different surgical treatments, including all covariates considered clinically significant and potentially influencing clinical outcomes comprising (1) age, (2) body mass index (BMI), (3) gender, (4) medical history, (5) decompression method, (6) preoperative clinical scores, (7) operative segments, and (8) smoking history.

**Table 1:** Inclusion and exclusion criteria

Inclusion criteria	Presence of low back pain, lower limb pain, or intermittent claudication
	Imaging showed single-segment lumbar spinal stenosis Failure of conservative treatment Good condition and can tolerate general anesthesia surgery
Exclusion criteria	Previous surgery at the lumbar spine Segment instability (spondylolisthesis > 5 mm or translation > 3 mm) Lumbar kyphosis or scoliosis deformity Combined with mental illness or spinal tumor Pregnant patients Incomplete follow-up information

### Surgical procedure

#### OSE approach

**Fig. 1:**
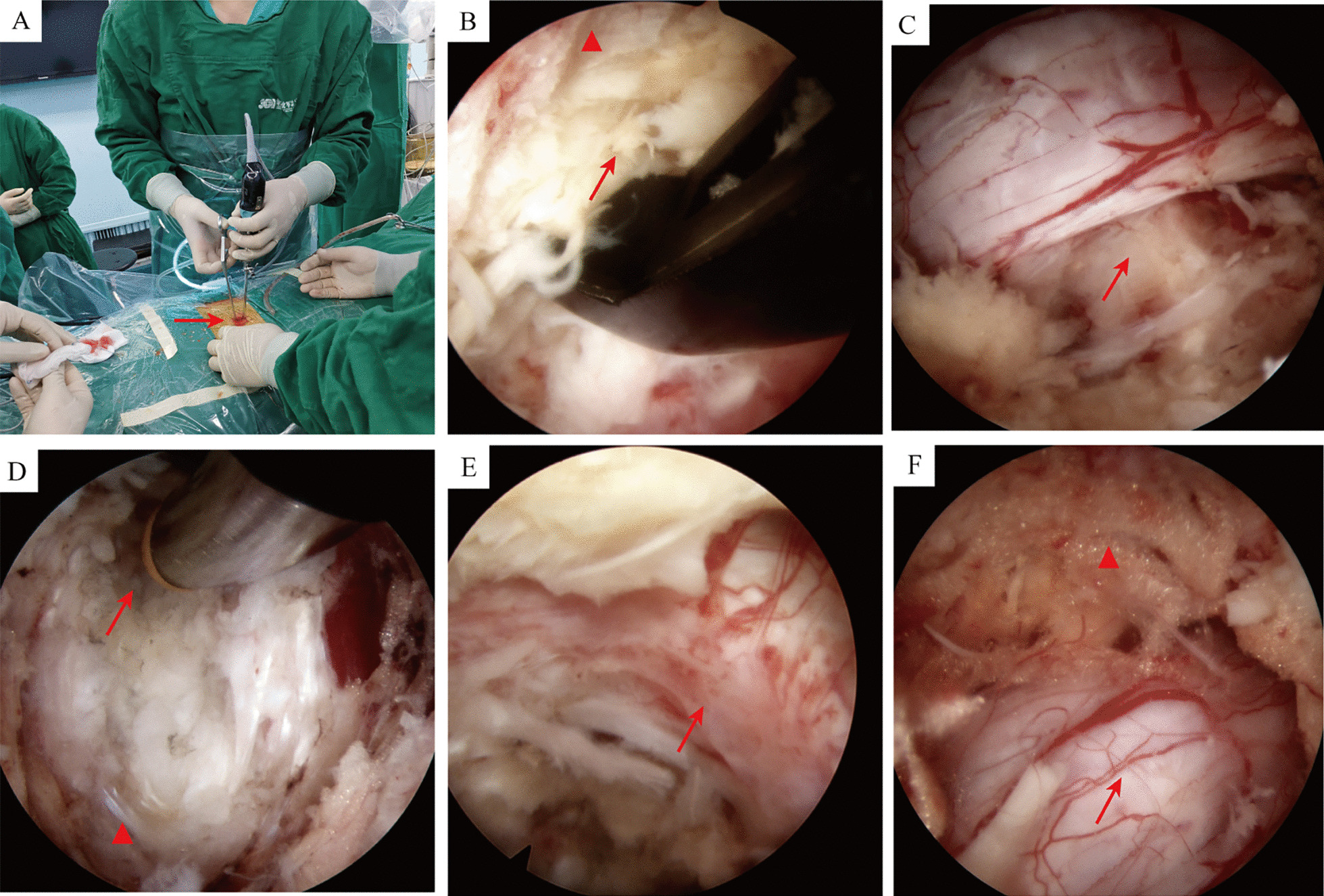
Endoscopy diagrams of the OSE technique. **A** Intraoperative operation by the attending surgeon, with both working and observation channels in the same soft incision (arrow); B Removal of a portion of the lamina (triangle) and the ligamentum flavum (arrow); **C** Herniated disc tissue (arrow) compressing the dural sac; **D** Incision of the annulus fibrosus (triangle) and removal of herniated disc tissue (arrow); **E** Nerve root (arrow) decompression; **F** Decompression of the dural sac(arrow), with the triangle for epidural fat

The patient was placed in the prone position under general anesthesia, and the target segment was localized under C-arm fluoroscopy. A longitudinal incision of approximately 2 cm was made 1.5 cm lateral to the intersection point of the responsible intervertebral space and the line connecting the spinous processes. A soft tissue dilator was applied to expand the soft tissue systematically to the bony surface of the lamina, and OSE endoscopy and operating instruments were subsequently placed. During the endoscopy, a plasma radiofrequency knife was used to remove the soft tissue to expand the surgical field, revealing the lower edge of the upper lamina, the upper edge of the lower lamina, ligamentum flavum, root of the spinous process, and medial border of the facet joints. Then, a high-speed dynamic grinding drill and a laminar rongeur were applied to resect the lower edge of the upper lamina and the upper edge of the lower lamina to the origin of the ligamentum flavum. The hypertrophied ligamentum flavum was simultaneously removed, exposing the nerve roots, dural sac, and intervertebral discs. For unilateral decompression cases, a nerve hook was applied to retract the nerve root and dural sac gently, and the herniated intervertebral disc and hypertrophic ossified posterior longitudinal ligament were thoroughly removed. Then, ventral decompression of the facet joint was performed to enlarge the nerve root canal. For bilateral decompression cases, decompression of the contralateral spinal canal and nerve root canal was primarily performed using the “over-top” technique, followed by ipsilateral decompression. The criteria for decompression were as follows: (1) a reduction in nerve root tension; (2) the absence of compressive tissue around the nerve; and (3) restoration of autonomous pulsation of the nerve root and dural sac (3). Finally, adequate hemostasis was achieved, and the endoscope was removed. Endoscopy diagrams are shown in Fig. 1.

#### UBE approach

Under C-arm fluoroscopy, the lower edge of the upper lamina of the responsible segment was localized, and two approximately 1.2 cm surgical incisions were made on the medial border of the ipsilateral pedicle, 1.5 cm above and below its horizontal line. Tissue dilation was performed systematically, and working and observation channels were placed. Afterward, the remaining procedures were the same as in the OSE approach.

**Fig. 2:**
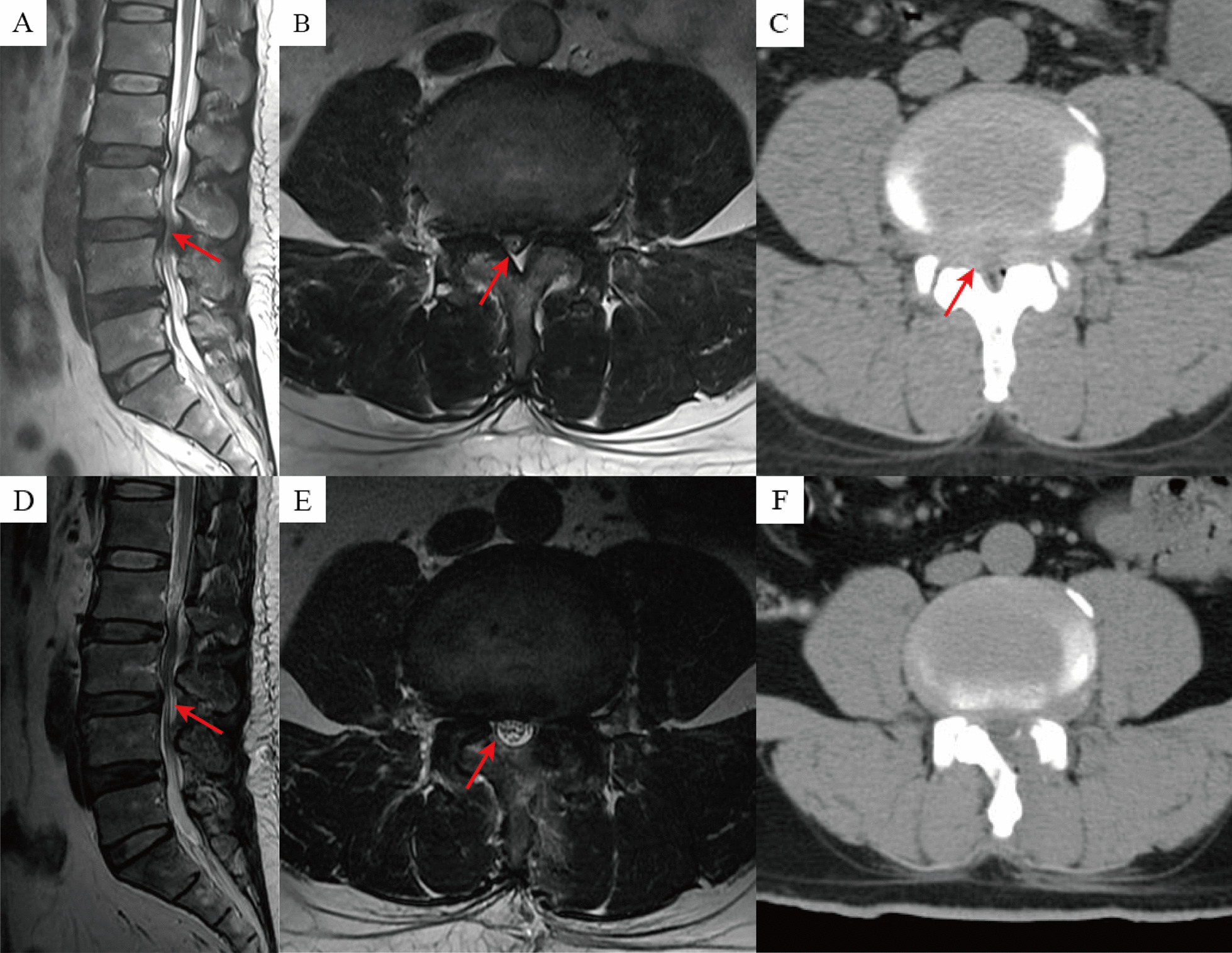
Images from a patient treated with OSE technique. **A**–**C** Preoperative MRI and CT showed L3-4 segment disc herniation with spinal stenosis; **D**–**F** Postoperative MRI and CT showed removal of the disc herniation, and adequate decompression of the spinal canal

**Fig. 3:**
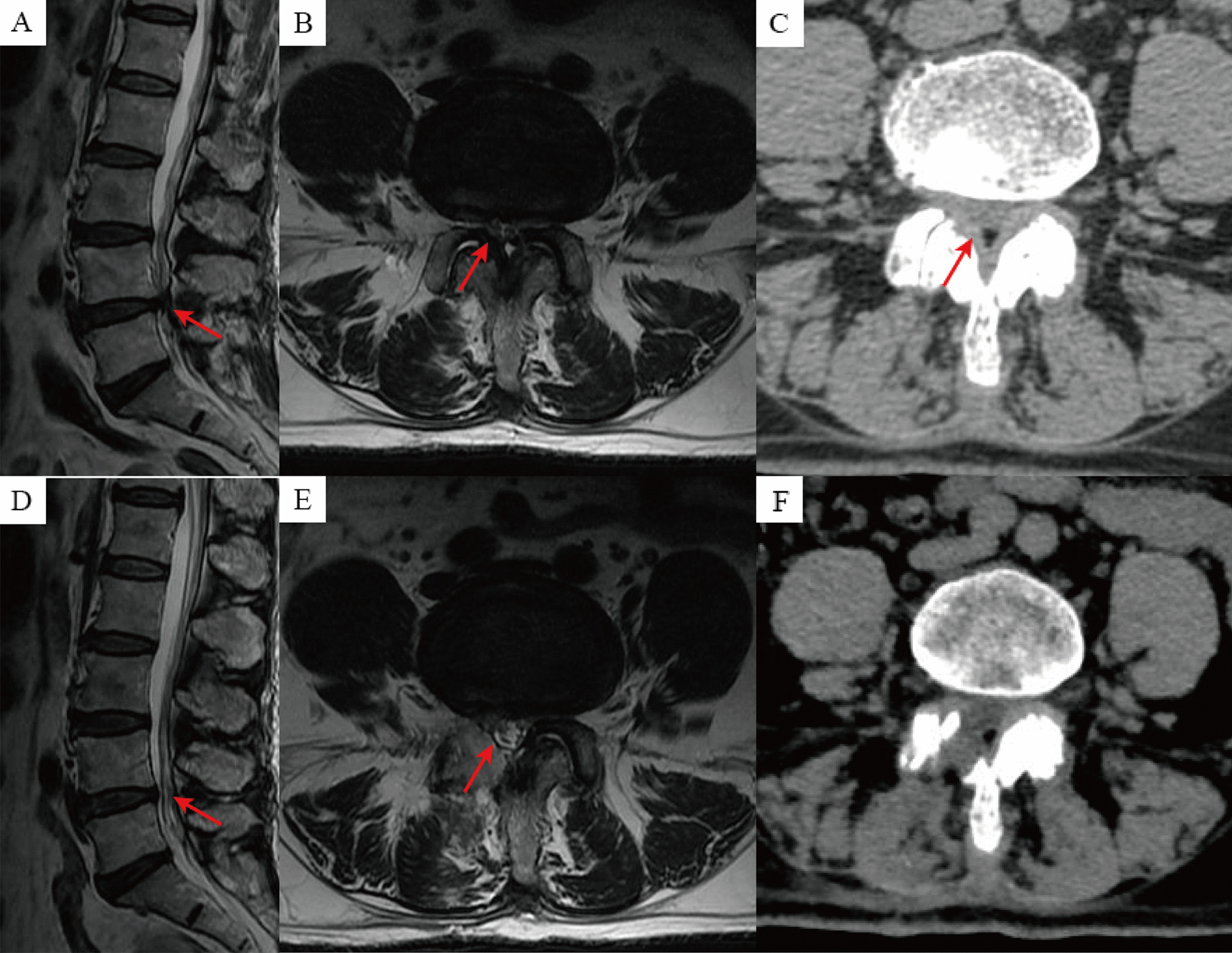
Images from a patient treated with UBE technique. **A**–**C** Preoperative MRI and CT showed L4-5 segment disc herniation with spinal stenosis; **D**–**F** Postoperative MRI and CT showed removal of the disc herniation, and adequate decompression of the spinal canal

Postoperatively, all patients were routinely managed with medications to prevent neural root edema and promote nerve nutrition. Patients were allowed to engage in appropriate activities using waist support on the second day postoperatively. They were instructed to wear the waist support for 4–6 weeks and to avoid vigorous exercise for 3 months. Representative cases in the OSE and UBE groups are shown in Figs. 2 and 3, respectively.

#### Data collection and measurement

Surgical outcomes of all successfully matched LSS patients were collected, including operative time, intraoperative blood loss, incision length, and hospital length of stay. The regular 1-year follow-up was conducted through telephone and/or email to record clinical and functional scores, imaging findings, and any potential complications.

#### Clinical assessment

Clinical and functional scores were determined using self-assessment questionnaires. Patients' clinical pain was assessed using the Visual Analog Scale of back pain (VAS-BP), leg pain (VAS-LP), and lumbar functional dysfunction was assessed using the Japanese Orthopedic Association score (JOA) and Oswestry Disability Index (ODI). At 12 months postoperatively, the modified MacNab criteria were recruited to evaluate patient satisfaction.

#### Imaging measurement

**Fig. 4:**
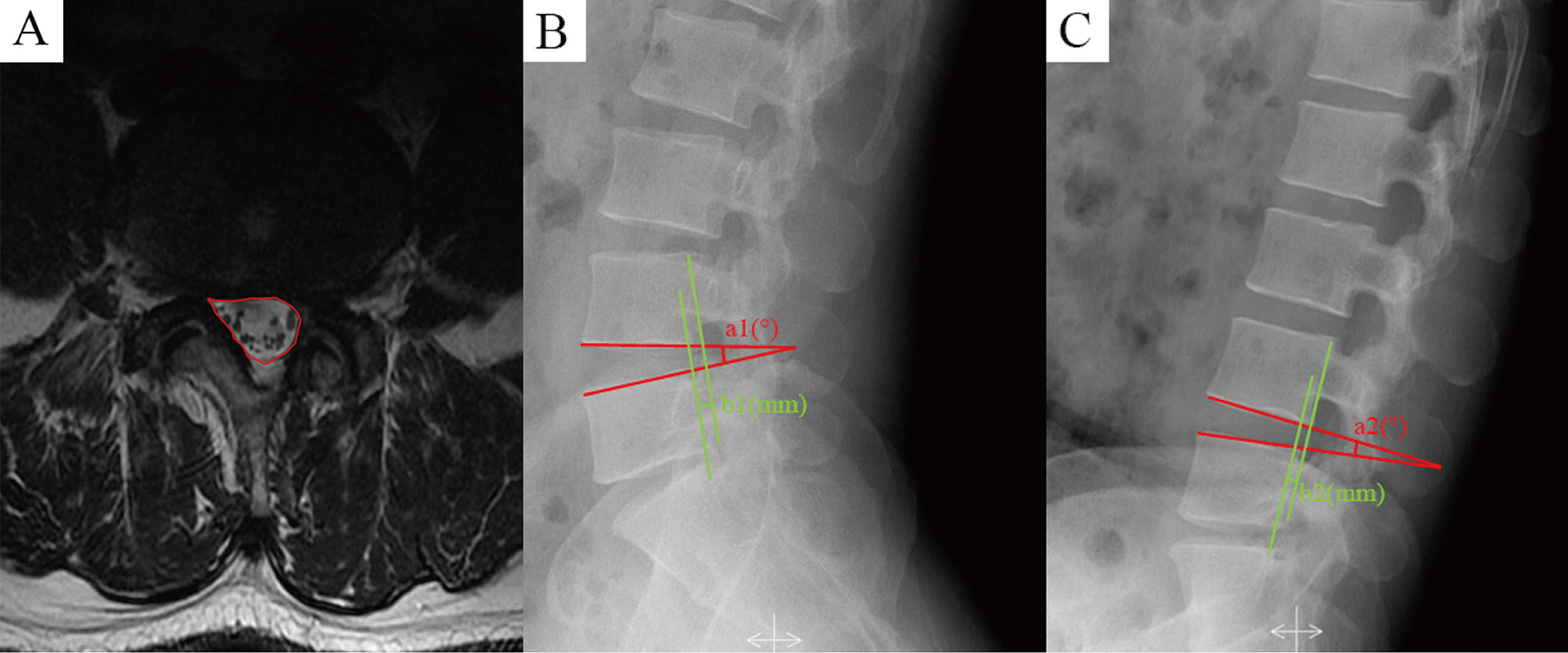
Schematic of imaging measurements. **A** The measurement of dural sac cross-sectional area (DCSA); **B**, **C** The measurement of range of motion (ROM) and sagittal translation (ST) in hyperextension and hyperflexion; ROM = a1–a2; ST =|b1–b2|

Imaging findings were measured using Image Viewer or AnyPacs software installed on workstations in DICOM or JPG format, including dural sac cross-sectional area (DCSA), lumbar range of motion (ROM), and sagittal translation (ST). DCSA was used to assess the degree of decompression of the spinal canal by both surgeries and DCSA improvement rate (postoperative DCSA—preoperative DCSA)/preoperative DCSA * 100%. ROM and ST were used to assess the impact of the two surgical approaches on lumbar spine stability. The following criteria were used to evaluate the lumbar instability: segment ROM ≥ 15° at L4-5 and above, ROM ≥ 20° at L5-S1, or ST > 3 mm [11, 12]. A schematic of the imaging measurements is shown in Fig. 4. All imaging findings were measured three times by three independent investigators and were averaged. In addition, we used previously reported computer vision and mathematical modelling methods to automatically measure the imaging data [13–15], and compared them with the manual measurement methods described above to verify the reliability of the data.

### Statistical analysis

All statistical analyses were performed using SPSS version 25 (IBM SPSS Statistics for Windows, Version 25.0. Armonk, NY: IBM Corp.). Student's t-test analysis was performed and described as means ± standard deviation (SD) for normally distributed continuous data. Within-group comparisons at different time points were analyzed using repeated measures analysis of variance. For non-normally distributed data, non-parametric tests were used. Categorical data were presented as frequencies and percentages (%) and were compared using the chi-square test. A p-value < 0.05 was considered statistically significant for differences between the two groups. In addition, the standardized mean difference (SMD) was used to assess the intergroup balance for given covariates [16]. SMD is unaffected by sample size and allows for comparing the relative balance of variables. According to Cohen's criteria, an SMD ≤ 0.2 indicates a slight difference in covariate balance [17]. Reliability of manual versus automated measurements were calculated using the intraclass correlation coefficient (ICC). According to Shrout and Fleiss, an ICC greater than 0.8 indicating strong agreement [18].

## Results

### Baseline characteristics before and after PSM

One hundred sixty patients with LSS were included before PSM, comprising 62 and 98 in the OSE and UBE groups, respectively. Covariates with SMD ≤ 0.2 and *P* > 0.05 were considered balanced and comparable between the two groups. However, we observed four covariates that were imbalanced in Table 2, including BMI (SMD = 0.282, *P* = 0.080), gender (SMD = 0.321, *P* = 0.047), VAS-LP score (SMD = 0.228, *P* = 0.169), and smoking history (SMD = 0.356, *P* = 0.028). After PSM, 104 LSS patients were included in the study, and the baseline characteristics of the two groups can be seen in Table 3, indicating that all covariates were well-balanced and comparable.

**Table 2:** Baseline characteristics before propensity score matching

Demographics	OSE group (n = 62)	UBE group (n = 98)	SMD	*P* value
Age (years)	61.55 ± 10.88	61.13 ± 10.33	0.040	0.862
BMI (kg/m^2^)	24.76 ± 2.89	23.92 ± 3.00	0.282	0.080
Gender, n (%)			0.321	0.047
Male	30 (48.39)	63 (64.29)		
Female	32 (51.61)	35 (35.71)		
Medical history				
Hypertension, n (%)	22 (35.48)	30 (30.61)	0.104	0.522
Diabetes, n (%)	15 (24.19)	26 (26.53)	0.053	0.741
Osteoporosis, n (%)	17 (27.42)	21 (21.43)	0.140	0.386
Decompression method			0.096	0.551
Unilateral decompression	39 (62.90)	57 (58.16)		
Bilateral decompression	23 (37.10)	41 (41.84)		
VAS-LP score	7.08 ± 1.01	7.32 ± 1.07	0.228	0.169
VAS-BP score	5.23 ± 1.06	5.28 ± 1.16	0.045	0.771
JOA score	12.03 ± 1.38	11.89 ± 1.43	0.100	0.480
ODI index	60.19 ± 10.39	61.39 ± 11.63	0.108	0.511
Responsible segment, n (%)			0.088	0.586
L4-5	35 (56.45)	51 (52.04)		
L5-S1	27 (43.55)	47 (47.96)		
Smoking, n (%)	11 (17.74)	33 (33.67)	0.356	0.028

**Table 3:** Baseline characteristics after propensity score matching

Demographics	OSE group (n = 52)	UBE group (n = 52)	SMD	*P* value
Age (years)	61.15 ± 10.14	60.81 ± 9.81	0.034	0.860
BMI (kg/m^2^)	24.27 ± 2.73	24.59 ± 2.93	0.113	0.560
Gender, n (%)			0.038	0.844
Male	29 (55.77)	28 (53.85)		
Female	23 (44.23)	24 (46.15)		
Medical history				
Hypertension, n (%)	18 (34.62)	17 (32.69)	0.041	0.836
Diabetes, n (%)	11 (21.15)	11 (21.15)	0.000	1.000
Osteoporosis, n (%)	13 (25.00)	10 (19.23)	0.138	0.478
Decompression method			0.040	0.839
Unilateral decompression	33 (63.46)	32 (61.54)		
Bilateral decompression	19 (36.54)	20 (38.46)		
VAS-LP score	7.21 ± 0.98	7.17 ± 1.06	0.039	0.854
VAS-BP score	5.21 ± 1.04	5.23 ± 1.26	0.017	0.987
JOA score	11.92 ± 1.34	11.98 ± 1.51	0.042	0.869
ODI index	60.88 ± 10.33	60.77 ± 12.14	0.010	0.958
Responsible segment, n (%)			0.039	0.842
L4-5	31 (59.62)	30 (57.69)		
L5-S1	21 (40.38)	22 (42.31)		
Smoking, n (%)	11 (21.15)	10 (19.23)	0.048	0.807

### Surgical results

All patients underwent surgery by the same surgical team. The OSE group had an average operative time of 62.42 ± 4.86 min, intraoperative blood loss of 51.83 ± 6.52 ml, incision length of 2.30 ± 0.14 cm, and hospital stay of 6.06 ± 0.87 days. The UBE group had an average operative time of 68.96 ± 4.56 min, intraoperative blood loss of 54.06 ± 8.13 ml, incision length of 2.70 ± 0.15 cm, and hospital stay of 6.13 ± 0.89 days. Table 4 demonstrates that the OSE group provides superior outcomes to the UBE group regarding operative time and incision length (*P* < 0.001). No statistically significant differences regarding intraoperative blood loss and hospital stay were achieved (*P* > 0.05).

**Table 4:** Comparison of surgical outcomes between the two groups

	OSE group (n = 52)	UBE group (n = 52)	*P* value
Operative time (min)	62.42 ± 4.86	68.96 ± 4.56	< 0.001
Intraoperative blood loss (ml)	51.83 ± 6.52	54.06 ± 8.13	0.192
Incision length (cm)	2.30 ± 0.14	2.70 ± 0.15	< 0.001
Hospital stay (d)	6.06 ± 0.87	6.13 ± 0.89	0.731

### Clinical evaluation

**Fig. 5:**
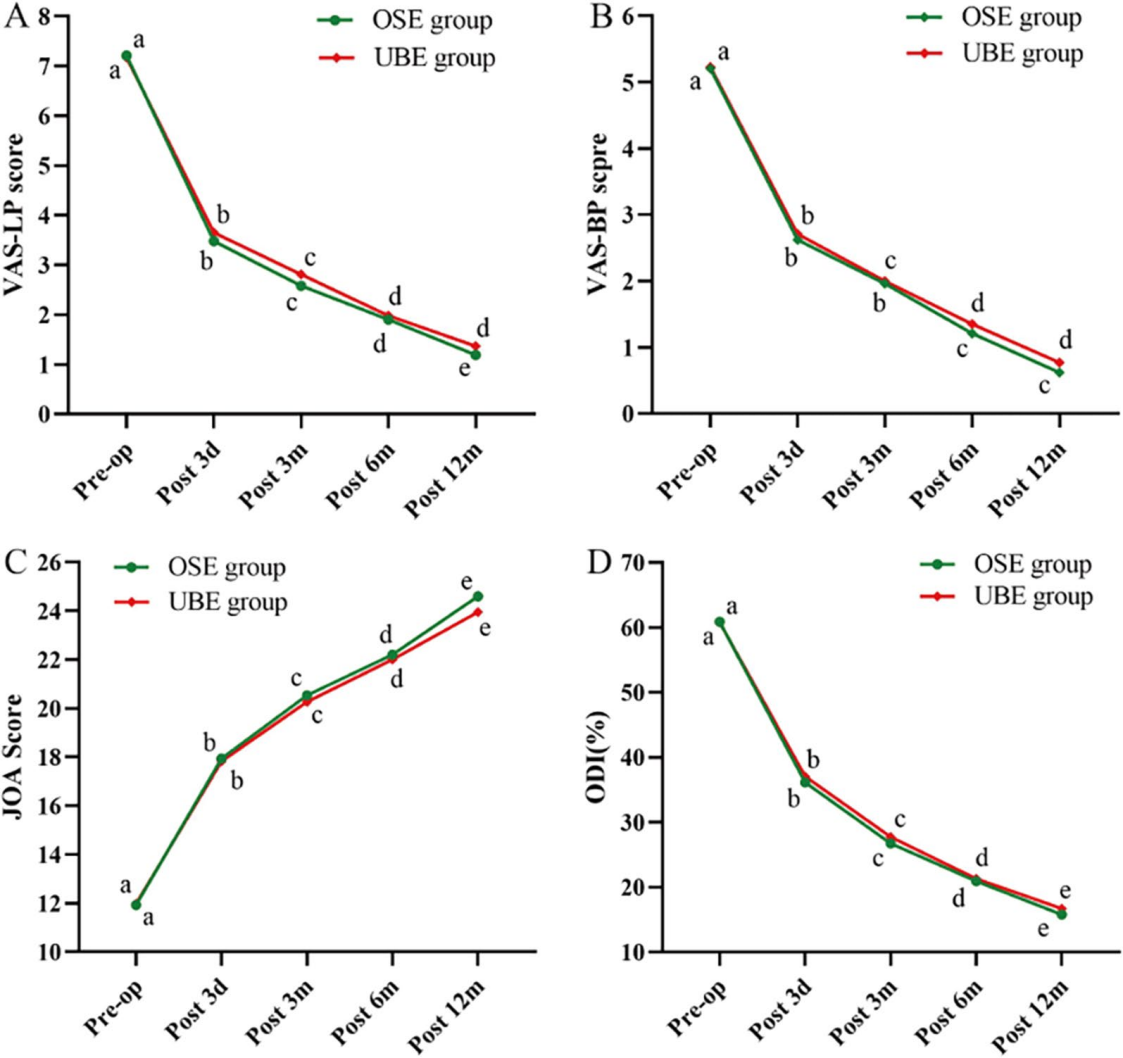
Results of clinical efficacy of functional scores. **A** Changes in VAS-LP scores over time. **B** Changes in VAS-BP scores over time. **C** Changes in JOA score over time. **D** Changes in ODI score over time. VAS-LP, Visual Analog Scale for leg pain; VAS-BP, Visual Analog Scale for back pain; JOA, Japanese Orthopedic Association; ODI, Oswestry Disability Index. **a-e** indicate the letter labelling of the time point difference (comparison within the group), if 2 time points have the same letter, there is no significant difference between the 2 time points (*P* > 0.05); otherwise, different letters at 2-time points mean the difference is significant (*P* ≤ 0.05)

The mean VAS-LP scores in the OSE group and UBE group decreased from 7.21 ± 0.98 and 7.17 ± 1.06 preoperatively (*P* = 0.854) to 3.48 ± 1.13 and 3.65 ± 1.30 at 3 days postoperatively (*P* = 0.592), 2.58 ± 1.16 and 2.81 ± 1.25 at 3 months postoperatively (*P* = 0.369), 1.90 ± 1.11 and 1.98 ± 1.18 at 6 months postoperatively (*P* = 0.866), and 1.19 ± 1.03 and 1.37 ± 1.22 at 12 months postoperatively (*P* = 0.602). There were no statistically significant differences in postoperative VAS-LP scores between the two groups (*P* > 0.05). Still, both groups showed significant improvement in postoperative VAS-LP compared to preoperative scores (*P* < 0.05) (Fig. 5A).

The mean VAS-BP scores showed a similar trend as the mean VAS-LP score, as shown in Fig. 5B. We found that the difference in VAS-BP scores between the two groups was not statistically significant (*P* > 0.05). However, both groups demonstrated significant improvement postoperatively (*P* < 0.05).

The mean JOA scores in the OSE group and UBE group significantly increased from 11.92 ± 1.34 and 11.98 ± 1.51 before operation (*P* = 0.869) to 17.94 ± 1.90 and 17.83 ± 2.22 at 3 days postoperatively (*P* = 0.906), 20.54 ± 2.26 and 20.27 ± 2.38 at 3 months postoperatively (*P* = 0.564), 22.21 ± 2.30 and 22.00 ± 2.29 at 6 months postoperatively (*P* = 0.755), and 24.60 ± 2.20 and 23.96 ± 2.43 at 12 months postoperatively (*P* = 0.302). There were no statistically significant differences in postoperative JOA scores between the two groups (*P* > 0.05), but both groups showed significant improvement in postoperative JOA scores compared to preoperative scores (*P* < 0.05) (Fig. 5C).

The mean ODI scores in the OSE and UBE group decreased from 60.88 ± 10.33 and 60.77 ± 12.14 before operation (*P* = 0.958) to 36.12 ± 9.46 and 37.04 ± 11.06 at 3 days postoperatively (*P* = 0.648), 26.73 ± 10.18 and 27.73 ± 11.17 at 3 months postoperatively (*P* = 0.634), 20.92 ± 8.71 and 21.31 ± 10.12 at 6 months postoperatively (*P* = 0.836), and 15.77 ± 8.80 and 16.69 ± 10.72 at 12 months postoperatively (*P* = 0.632). There were no statistically significant differences regarding postoperative ODI scores between the two groups (*P* > 0.05); however, both groups showed significant improvement in postoperative ODI scores (*P* < 0.05) (Fig. 5D).

According to the modified MacNab criteria, there were 29 cases of excellent, 20 cases of good, 3 cases of fair, and 0 cases of poor in the OSE group, with an excellent and good rate of 94.23%. There were 26 cases of excellent, 21 cases of good, 5 cases of fair and 0 cases of poor in the UBE group, with an excellent and good rate of 90.38%. At 12 months postoperatively, the two groups revealed no statistically significant difference regarding the excellent and good rate (*P* = 0.713).

### Imaging measurements

The imaging outcomes of the two groups are shown in Tables 5 and 6. There were no statistically significant differences regarding DCSA between the OSE and UBE groups (*P* > 0.05); however, both showed significant postoperative improvement (*P* < 0.001). At 12 months postoperatively, the improvement rate of DCSA in the OSE and UBE groups were 121.42 ± 22.45% and 124.06 ± 19.76% for manual measurement (*P* = 0.263), and 113.37 ± 17.43% and 116.72 ± 17.32 for automatic measurement (*P* = 0.280), respectively. Postoperative ROM and ST were within the normal range (ROM of the L4-5 segment was < 15°, ROM of the L5-S1 segment was < 20° and ST was < 3 mm), and no lumbar instability was recorded. Similarly, no statistically significant differences regarding ROM and ST were found between the two groups (*P* > 0.05). In Table 7, we observed that the ICC for DCSA, ROM, and ST were all greater than 0.8 (0.844–0.951), indicating strong agreement between the imaging data measured by the two methods.

**Table 5:** Comparison of manually measured imaging outcomes between the two groups

	OSE group (n = 52)	UBE group (n = 52)	*P* value
DCSA (mm^2^)			
Pre-op	75.90 ± 11.77	76.13 ± 11.85	0.922
3 days	169.33 ± 13.37	172.45 ± 14.99	0.265
12 months	165.66 ± 12.80	168.37 ± 14.25	0.310
ROM (°)			
Pre-op	8.53 ± 1.47	8.42 ± 1.24	0.687
3 days	8.89 ± 1.49	8.79 ± 1.28	0.718
12 months	8.58 ± 1.52	8.45 ± 1.26	0.633
ST (mm)			
Pre-op	1.37 ± 0.20	1.35 ± 0.22	0.653
3 days	1.41 ± 0.21	1.39 ± 0.22	0.596
12 months	1.46 ± 0.21	1.44 ± 0.23	0.617

**Table 6:** Comparison of automatically measured imaging outcomes between the two groups

	OSE group (n = 52)	UBE group (n = 52)	*P* value
DCSA (mm^2^)			
Pre-op	81.43 ± 11.67	81.46 ± 11.79	0.876
3 days	175.65 ± 14.51	178.05 ± 15.33	0.414
12 months	171.94 ± 14.31	174.66 ± 14.82	0.344
ROM (°)			
Pre-op	9.07 ± 1.47	8.98 ± 1.27	0.740
3 days	9.55 ± 1.50	9.47 ± 1.29	0.777
12 months	8.99 ± 1.48	8.87 ± 1.24	0.665
ST (mm)			
Pre-op	1.48 ± 0.20	1.47 ± 0.22	0.812
3 days	1.53 ± 0.20	1.52 ± 0.23	0.885
12 months	1.58 ± 0.21	1.58 ± 0.24	0.976

**Table 7:** Comparison of ICC between the two measurement methods

	ICC	95%CI
DCSA		
Pre-op	0.892	− 0.020–0.975
3 days	0.914	− 0.011–0.981
12 months	0.900	− 0.017–0.977
ROM		
Pre-op	0.917	− 0.008–0.982
3 days	0.893	− 0.002–0.977
12 months	0.951	− 0.017–0.989
ST		
Pre-op	0.862	− 0.016–0.968
3 days	0.844	− 0.015–0.964
12 months	0.848	− 0.018–0.965

### Complications

The overall complication rate in OSE and UBE was 1.92% and 5.77%, respectively. One patient experienced a dural tear in the OSE group. Two patients experienced a dural tear, and one patient had a postoperative epidural hematoma in the UBE group. The symptoms of complicated patients were relieved after conservative treatment. There was no statistically significant difference regarding complications between the two groups (*P* = 0.610). No severe complications, such as intervertebral space infection or nerve root rupture, were noted during the follow-up period.

## Discussion

LSS is a common cause of disability in the elderly, affecting approximately 103 million individuals worldwide, with a prevalence increasing with age [19]. LSS represents the most common indication for spinal surgeries. There is currently solid literature, including a long-term randomized trial confirming that approximately 80% of LSS patients could achieve favorable clinical outcomes following surgical management [20]. With advancements in surgical instruments and endoscopic techniques, minimally invasive spine surgery has been considered an alternative to traditional open surgery for the treatment of LSS, offering advantages including less trauma, faster recovery time, and shorter hospital length of stays, which is more in line with the concept of enhanced recovery after surgery (ERAS) [4, 6]. The key to treating LSS is to achieve sufficient dural sac nerve roots and dural sac decompression while minimizing the loss of the lumbar vertebral structures [21]. Previous biomechanical studies have demonstrated that the posterior lumbar column, such as the facet joints and joint capsules, plays a vital role in maintaining spinal stability [22].

In 1996, De Antoni et al. [23] first described the UBE decompression technique, and Choi et al. [24] first applied the UBE technique for treating LSS in 2016, reporting satisfactory clinical outcomes. Since then, UBE has been widely developed as an effective minimally invasive surgical technique for treating LSS, demonstrating unique clinical and technical advantages. Compared to traditional open surgery, studies have shown that UBE for LSS can achieve comparable clinical outcomes. It could provide superior outcomes regarding length of hospital stay, intraoperative blood loss, and paravertebral muscle loss [3, 25]. Compared with coaxial endoscopy, the UBE technique has independent working and observation channels, providing greater surgical flexibility and a wide field of view, resulting in comprehensive spinal canal decompression [26, 27]. In addition, the UBE technique has no restrictions on decompression surgical instruments, which significantly enhances decompression efficiency [8, 28]. The relatively smooth learning curve of the UBE technique is also one rationale behind its administration [21, 29]. From the illustration point of view, the UBE technique and conventional methods share similar characteristics, and both utilize the principle of the arthroscopic triangle, making it easier for beginners to learn.

The OSE technique is an innovative advancement based on the UBE technique and a continuation of coaxial endoscopy, demonstrating new clinical advantages. Like the UBE technique, the OSE consists of working and observation channels; however, these two channels are located within the same soft incision, allowing independent and free operation of the endoscope and instruments through a single port [9]. The OSE technique also imposes no surgical instrument-related limitations and keeps similar advantages to the UBE technique, such as flexible operation, a wide field of view, and efficient decompression. Besides, it does not have the limitation of forming a “V” angle and allows for parallel operations, effectively avoiding the blind spots in the field of view imposed by the UBE technique. It also reduces the risk of nerve root and dural sac injury, especially in patients with narrow intervertebral spaces. In addition, the OSE technique performs decompression within the same incision, which effectively avoids the likelihood of instrument misplacement due to complex positioning during UBE surgery and makes hemostasis relatively more comfortable in endoscopic surgeries. Less experienced physicians may face collisions between the endoscope and instruments during the surgery. However, corresponding issues could be avoided with proficient practice. In cases with severe soft tissue adhesions involving the nerve roots and dural sac, the decompression process of OSE technique should be operated cautiously to avoid nerve root and dural sac injuries caused by the wide traction of soft tissues with conventional open instruments. For surgeons with rich clinical experience in UBE techniques and coaxial endoscopy, the OSE technique can be more proficient in treating LSS. Furthermore, the OSE technique brings a more open surgical experience to endoscopic surgery; however, it is minimally invasive. For physicians with a foundation in both open and minimally invasive surgery, it is rational to learn and master the technique more quickly.

In our study, no significant difference was observed regarding VAS-LP, VAS-BP, JOA, and ODI scores between the OSE and UBE techniques for treating LSS patients. The clinical scores of all patients significantly improved after surgery. Therefore, both surgical approaches for treating LSS may yield satisfactory clinical outcomes, leading to significant relief of the patient's pain and improving neurological function. Despite the similar clinical efficacy of both techniques, they still have distinct characteristics in treating LSS patients. Compared to the UBE technique, the OSE provides a shorter operative time, smaller incisions, and less accompanied trauma. Under general anesthesia, prolonged operative time is an essential factor contributing to delayed patients' postoperative resuscitation [30] and could increase the risk of surgical site infection [31]. Adequate decompression of the spinal canal and favorable postoperative stability of the lumbar spine are key factors influencing the prognosis of LSS patients. In our study, both groups showed a significant postoperative increase in DCSA without compressing the dural sac and nerve roots. The results indicate that both techniques could achieve sufficient spinal canal decompression, which can contribute to alleviating the neurological function and symptoms of patients. The extent of facet joint resection during surgery is an essential factor affecting spinal stability. Currently, most spine surgeons accept the notion that removing more than 50% of the facet joints during lumbar decompression significantly disrupts the biomechanical stability of the spine [32, 33]. In the current study, ROM and ST of all operative segments were in the normal range within 1 year postoperatively, and no lumbar instability was noted. However, it could not be concluded that the OSE and UBE techniques may not lead to lumbar instability. The limited view under endoscopy requires the surgical operator to possess a rich understanding of anatomy and clinical experience to balance the extent of facet joint resection and adequate spinal canal decompression effectively.

All patients underwent successful surgery under endoscopy without any case converted to open surgery. In the OSE group, one patient experienced a dural tear. In the UBE group, two patients experienced a dural tear, and one patient had a postoperative epidural hematoma. By reviewing and analyzing the cases, the dural tears were considered to be caused by the severe adhesions between the ligamentum flavum and the dural sac, which resulted in the tearing of the dura sac during the decompression procedure. The epidural hematoma was mainly caused by incomplete intraoperative hemostasis. Therefore, preoperative individualized decompression plans are required based on the patient's symptoms and imaging findings. Meanwhile, precise targeted decompression, gentle manipulation of the nerve roots and dural sac, and careful hemostasis are required to ensure a clear operative field during surgery and prevent postoperative complications. Additionally, continuous intraoperative saline irrigation can reduce the risk of infection in endoscopic procedures [34]. No severe complications, such as intervertebral space infection or nerve root rupture, were recorded.

There are some limitations in the current study. Firstly, it was retrospectively designed with an inevitable imbalance of confounders between the included groups. Although we used PSM methods for post-hoc randomization and applied SMD for between-group balancing, selection bias may still exist. Secondly, despite the imaging results were measured manually by independent reviewers or automatically measured by computer vision and mathematical modelling, the measurement errors still could not be completely avoided. Thirdly, the small number of cases and the short follow-up warrants further research and exploration.

## Conclusion

OSE is an alternative technique to UBE for the treatment of LSS, with similar satisfactory clinical outcomes, shorter operative time, and smaller incision length. Further studies are needed for long-term efficacy.

## Data Availability

The datasets used during the current study are available from the corresponding author on reasonable request.
